# Family Health Climate and Adolescents’ Physical Activity and Healthy Eating: A Cross-Sectional Study with Mother-Father-Adolescent Triads

**DOI:** 10.1371/journal.pone.0143599

**Published:** 2015-11-25

**Authors:** Christina Y. N. Niermann, Stef P. J. Kremers, Britta Renner, Alexander Woll

**Affiliations:** 1 Institute of Sports and Sports Science, Karlsruhe Institute of Technology, Karlsruhe, Germany; 2 Department of Health Promotion, Maastricht University, Maastricht, the Netherlands; 3 Department of Psychology, University of Konstanz, Psychological Assessment and Health Psychology, Konstanz, Germany; Vanderbilt University, UNITED STATES

## Abstract

**Introduction:**

The importance of the family environment for children’s and adolescents’ health behavior has been demonstrated, the underlying mechanisms of this influence remain unclear. Therefore, the aim of the study was to investigate the relationship between family environmental and individual determinants. It was hypothesized that the Family Health Climate (FHC) is associated with adolescents’ physical activity and dietary behavior and that intrinsic motivation mediates this association.

**Methods:**

Cross-sectional data were collected from 198 families (mother, father, and child) using questionnaires. Perceptions of FHC of mothers, fathers, and their children were assessed using the FHC-scales for physical activity (FHC-PA) and nutrition (FHC-NU). The adolescents also rated their intrinsic motivation for exercise and healthy eating, their physical activity and consumption of healthful food. A structural equation model was analyzed and a bootstrapping procedure was used to test direct and indirect effects.

**Results:**

The FHC-PA was related to the amount of weekly physical activity and the FHC-NU to the consumption of fruit, vegetables and salad. These effects were mediated by adolescents’ intrinsic motivation; the indirect effects were significant for both behaviors.

**Discussion:**

These results emphasize the importance of the FHC in shaping adolescents’ physical activity and dietary behavior. Individual motivational factors are potential mediators of family and parental influences. Considering family-level variables and their interaction with individual factors contributes to the understanding of adolescents’ health behavior.

## Background

Previous research has shown that, on average, children and adolescents are not sufficiently physically active [1] and frequently make unhealthy food choices [2, 3]. These behaviors have been associated with negative effects on health and well-being [1, 4, 5]. These facts provide compelling evidence that–even after decades of research on determinants of health behavior–we still need to identify effective determinants and mechanisms underlying the behavior patterns of children and adolescents.

**Fig 1:**
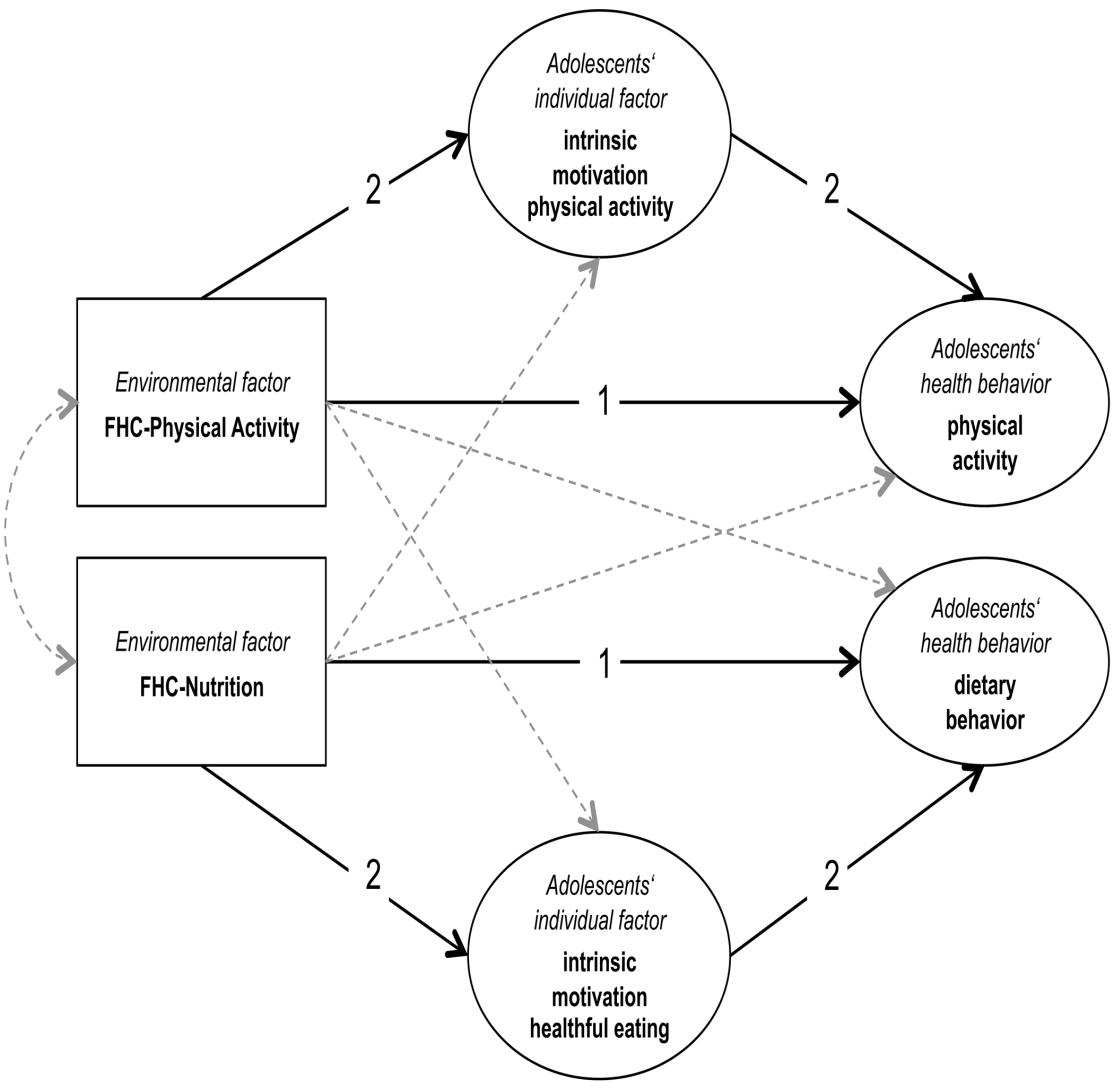
Conceptual model of family health climate, intrinsic motivation and adolescent health behavior.

The objective of this study was to determine the interrelationship of individual and family environmental factors affecting adolescents’ physical activity and dietary behavior. Specifically, we propose a mediational model where the influence of environmental factors on individual health behavior is mediated through individual factors. This model builds the conceptual framework of this study (see Fig 1).

### Determinants of adolescents health behavior

Individual behavior is affected by both individual and environmental determinants and their interactions [6, 7]. Adolescents’ health behavior is embedded in social contexts, for example peer groups, school or family environment. Especially the family is important because of its lasting effect on children’s and adolescents’ physical activity and dietary behavior [8–10].

#### Environmental determinant: the family

Many studies on family environmental influences have focused on the parent-child relationship and the influence of parental behaviors. Previous research demonstrated the important role of parents in the development of a healthy lifestyle in their children [11, 12]. Parents are gatekeepers of their children’s healthful eating and engagement in physical activities. Influencing factors include parenting practices such as modelling, monitoring, support, and encouragement [13–17] and more general concepts such as parenting styles [18–20]. However, inconsistent results regarding the influence of general parenting on children’s health behaviors have been reported [21, 22].

Other studies have examined more specific aspects of the family environment including media equipment, physical activity equipment, availability of healthful foods, and quantity and quality of family meals [23–28]. Although these studies emphasized the importance of the family environment for children’s and adolescents’ health behavior, the relationship between the different family environmental determinants and the underlying mechanisms of influence remain unclear [29, 30], and a theoretical framework is often missing. Furthermore, parental behaviors as well as specific environmental factors do not reflect the family as a whole [31].

***The Model of Family Reciprocal Determinism*:** According to theoretical approaches such as Bandura’s Social Cognitive Theory, environmental and individual determinants are reciprocally interrelated and both affect health behavior [6]. The Model of Family Reciprocal Determinism extends the reciprocal determinism from the individual to the family and provides a framework for describing influences of the family environment on individual health behavior [32].

The family members live and interact in a shared family environment. Individuals as well as interactions between them affect the family environment, which in turn affects the individuals and their interactions. Hence, the ‘family-level’ extends beyond the individuals and their shared environment. The family can hence be considered a system [31]. According to the family systems approach, family is more than the sum of the individuals.

This theoretical framework implies reciprocal influences within and between individuals as well as between the individuals and the shared family environment. Hence, attributes of the family environment should affect individuals’ behavior and individual determinants. One relevant aspect of the family environment may be the Family Health Climate [33].

***Family Health Climate*:** The Family Health Climate (FHC) is defined as the shared perceptions and cognitions concerning a healthy lifestyle within a family. It reflects the individual experience of daily family life, the evaluation of health-related topics and expectations with respect to typical values, behavior routines and interaction patterns within the family. The FHC serves as a framework for an individual’s everyday health behavior, is the basis of regulating health-related behaviors and provides references for valuing and interpreting individuals’ own behavior and that of others. Hence, the FHC is an aspect of the family environment that shapes everyday health behaviors of the family members. The FHC can be evaluated regarding healthy eating using the FHC-Nutrition scale and regarding physical activity using the FHC-Physical Activity scale. The FHC represents a family level variable that is intra- and inter-individually correlated to family environmental and to individual factors [33].

Previous research indicated that physical activity and dietary behavior are interrelated [34–38]. Because physical activity and eating take place within the family environment and are inherent parts of everyday family life, studying determinants of physical activity and dietary behavior simultaneously will be superior to studying these factors in isolation [39]. This allows examining the relationship between these two behavioral domains (see Fig 1, cross behavioral paths are presented by dotted lines). In particular, it enables testing for commonalities in form of shared variance and differences in form of unique variance.

In addition to environmental determinants of adolescent physical activity and eating behavior, individual’s motivation is well known to play a crucial role in the initiation and the maintenance of physical activity and healthful eating [40].

#### Individual determinant: intrinsic motivation

Besides the quantity of motivation that predicts the engagement in different types of behaviors, it is also the ‘quality’ of motivation–intrinsic vs. extrinsic–that matters [41]. Previous studies have shown that autonomous motivation is an important predictor of initiating and maintaining healthful behaviors such as regular physical activity and a healthy diet. Intrinsically motivated behaviors are adopted because of interest and enjoyment [42] and thus are more likely to be maintained [43, 44]. Intrinsic motivation depends on the satisfaction of three basic psychological needs: autonomy, relatedness, and competence [41]. Individual’s motivation and the satisfaction of these basic needs are embedded in a social context. The social contexts affect need satisfaction by either facilitating or impairing the satisfaction of the basic needs and the development and maintenance of intrinsic motivation [42].

Hence, we hypothesized that the family environment affects adolescent’s intrinsic motivation [45], which in turn influences their health behaviors (Fig 1).

#### Family Health Climate, intrinsic motivation and health behavior

The FHC is a family level variable reflecting an aspect of the shared family environment. Due to the reciprocal influences between the shared family environment and the individuals and their interactions, the FHC should be associated with individuals’ cognitive, motivational, and behavioral variables, with interactions related to physical activity or nutrition within the family, and with routines in family life. A positively evaluated FHC reflects that both being physically active and eating healthy is an important and integral part of everyday family life.

One of the core assumptions of the Self-Determination Theory is that children naturally tend to internalize attributes of the social context such as values, attitudes, and beliefs and to integrate them into a coherent sense of self [42, 45]. Therefore, it could be assumed that living in a family with a positive FHC is associated with autonomous motivation to be physically active and to choose healthful foods, which should be associated with more healthful behavior.

### Aims and hypotheses

The aim of this study was to determine the relationship between FHC, adolescents’ intrinsic motivation and adolescents’ physical activity and dietary behavior. Specifically, the following hypotheses were tested using cross-sectional data from mother-father-adolescent triads (see paths in Fig 1):

1. The FHC-Physical Activity is associated with adolescents’ physical activity and the FHC-Nutrition is associated with adolescents’ consumption of healthful foods.
2. FHC-Physical Activity has an indirect effect on physical activity via intrinsic motivation to engage in physical activities, and FHC-Nutrition has an indirect effect on adolescents’ consumption on healthful food via intrinsic motivation to choose healthful foods.

## Methods

### Procedure

Participants were recruited from 11 secondary schools in the district of Konstanz, Germany. Students from the seventh year upwards (corresponding to an age of at least 12 years) were approached. After making an appointment with the schools’ principal the classes chosen by the principal were visited. The students were informed about the aims and requirements of the ‘Family and Health-Study’ and received an envelope with three questionnaires: one for themselves, one for their mothers, and one for their fathers. In total, 1500 students were approached. The students were asked to forward the questionnaires to their parents and were informed that each person needed to complete the questionnaire individually. Within one week, the students and their parents returned the completed questionnaires in sealed envelopes to their class teacher who gave them to the principal where the envelopes were collected by a member of the research team.

### Ethics statement

According to the German Research Foundation [46] and the National Science Foundation [47] this research is exempt from institutional review board review. The study included an anonymous survey that did not involve collection of identifiable data. The survey was purely observational (non-invasive, non-interactive) and did not induce any type of psychological stress or anxiety. The participants were not member of a vulnerable group.

The researchers visited the classes and briefly introduced the study to the students and the teachers. The students received three questionnaires (one for themselves, one for their mothers, and one for their fathers), detailed information regarding voluntary participation, handling of the questionnaires and processing of their data and an envelope. Written informed consents were obtained from the parents of the participating students. The written consent and the completed questionnaires were returned within the sealed envelope to the teachers. Apart from this, there was no interaction between the researchers and the participating students or parents. The participants had the opportunity to opt out of the study until the sealed envelopes were returned to the teachers. Thereafter, it was no longer possible to assign the envelope to a family.

The study protocol was defined by a multidisciplinary expert panel of scientists involved in the EATMOTIVE project. The study fully conformed to the Declaration of Helsinki and the ethics guidelines of the German Psychological Society. Students and parents received detailed information regarding voluntary participation, handling of the questionnaires and processing of their data according to the ethics guidelines of the German Psychological Society [48].

### Participants

Three-hundred and nineteen families filled in at least one questionnaire. More than 80% of these families were two parent families (n = 253), while nearly 17% were single parent families (n = 53). From seven families there was no information available regarding family structure. According to the aims and hypotheses presented in this study, families where child, mother, and father completed the questionnaires and lived in the same household were included in the analyses (N = 198; 62.1%). The children had a mean age of 14.0 years (SD = 1.2 years), 60.6% were female. More than 70% (n = 122) of the adolescents attended the highest level of secondary school (‘Gymnasium’ in the German tripartite school system). The mothers had a mean age of 45.1 years (SD = 4.2 years; range 34 to 56 years). Sixty-two (31.3%) mothers had a university-entrance diploma (‘Abitur’) and 21 (10.6%) had an advanced technical college certificate (‘Fachhochschulreife’). At the time of the study, 25 (12.6%) mothers worked full-time, 139 (70.2%) worked part-time, 2 (1%) were unemployed or retired, 2 (1%) were on parental leave, 21 (1.6%) were homemakers and 8 (4.0%) were freelancers. The fathers had a mean age of 47.7 (SD = 6.5 years, range 27 to 74 years). Seventy-seven (39.1%) fathers had a university-entrance diploma and 38 (19.3%) had an advanced technical college certificate. At the time of the study, 177 (89.4%) fathers worked full-time, 6 (3.0%) worked part-time, 1 was on parental leave, 6 (3.0%) were unemployed or retired, 4 (2.0%) were homemakers and 4 (2.0%) were freelancers.

### Measures

#### Demographics

Mothers, fathers and adolescents completed questionnaires to obtain demographic information including age, gender, educational level and family structure. Age and gender were assessed by single questions. Adolescents stated which school they attended and which persons lived currently in the household (categories: ‘mother’, ‘father’, ‘siblings’, ‘grandmother’, ‘grandfather’ and ‘others’). Mothers and fathers stated their marital status, which was categorized into ‘living alone’ and ‘living in a partnership/marriage in the same household’ and ‘living in a partnership/marriage not in the same household’.

Parents’ educational level was assessed by asking for the highest school qualification. According to the German tripartite school system the categories ranged from ‘no qualification’ to ‘university-entrance diploma’. Employment status was categorized in ‘full-time’, ‘part-time’, ‘on parental leave’, ‘homemakers’, ‘unemployed’, ‘retired’, and ‘freelancer’.

#### Family Health Climate

The Family Health Climate was assessed with the FHC-Scales for physical activity (FHC-PA) and for nutrition (FHC-NU) using a validated questionnaire [33]. The FHC-PA Scale consists of 14 items pertaining to three subscales (value, e.g. ‘In our family it is normal to be physically active in our leisure time’; cohesion, e.g. ‘…we have fun doing physical activities together (e.g. bike tours, hikes)’, and information, e.g. ‘…we collect information (e.g. on the internet) on physical activity and exercise’). The FHC-NU Scale comprises 17 items pertaining to four subscales (value, e.g. ‘…it is normal to choose healthful foods’, cohesion, e.g. ‘…we appreciate spending time together during meals’, communication, e.g. ‘…we talk about which foods are healthful’, and consensus, e.g. ‘…we rarely argue about food- or diet-related matters’). The items were rated on a 4-point Likert-type scale ranging from 0 = ‘not true’ to 3 = ‘true’. Scores representing the mean of all items were calculated for the FHC-PA and FHC-NU, respectively. Mothers, fathers and adolescents each completed the scales individually. The internal consistencies in this study were α_FHC-PA_ = .92 and α_FHC-NU_ = .86 for mothers, α_FHC-PA_ = .90 and α_FHC-NU_ = .86 for fathers, and α_FHC-PA_ = .90 and α_FHC-NU_ = .85 for adolescents. The Intra Class Correlations (ICC) were calculated to determine the agreement between the ratings of mothers, fathers and children. The ICC’s for FHC-PA were ICC (1,1) = .51 and ICC (1,3) = .75 and for FHC-NU ICC (1,1) = .29 and ICC (1,3) = .56. The overall FHC was calculated as the sum of the individual scores of child, mother and father (FHC_agg_). The FHC_agg_ scores ranged between 0 and 9 and reflect the climate score within the family across its members [49].

#### Intrinsic motivation

Adolescents’ intrinsic motivation of engaging in physical activities was measured by the subscale ‘intrinsic regulation’ of an adapted version of the Behavioural Regulation of Exercise Questionnaire 2 (BREQ-2) [50]. The subscale comprises four items (e.g. ‘I exercise because it’s fun’). The responses were scored on a 4-point Likert-type scale ranging from 0 = ‘not true’ to 3 = ‘true’. The internal consistencies in the current study were good (α = .90).

Adolescents’ intrinsic motivation of healthful eating was measured using an adapted version of the ‘intrinsic regulation’ subscale of the Regulation of Eating Behavior Scale (REBS) [51]. Item wording was modified to represent adolescents’ language and conditions of adolescents’ eating behavior. The focus of the items was changed to liking healthful meals and feeling good when eating healthful meals (four items: ‘I just like eating healthful meals’, ‘Eating healthful foods makes me feel good’, ‘I enjoy eating healthful food’, ‘I just feel good when I eat healthful food’). Responses were scored on the same 4-point Likert-type scale as described above, with a good internal consistency in this study (α = .87).

Both scales (BREQ-2, REBS) were translated and back-translated by a native speaker with excellent German skills and four German researchers with excellent English skills. Discrepancies were resolved through discussion.

#### Frequency of physical activity

Adolescents completed two screening items to assess their habitual physical activity (60-minute MVPA screening measure) [52]. Corresponding to the guideline of the World Health Organization [53] the number of days with at least 60 minutes of moderate-to-vigorous physical activity during a ‘normal week’ and during the ‘last week’ were captured (‘In the past 7 days, on how many days were you physically active for a total of at least 60 minutes per day?’ and ‘In a typical or usual week, on how many days are you physically active for a total of at least 60 minutes per day?’). The 60-minutes MVPA screening measure is a brief, reliable and valid method for assessing adolescent physical activity [52]. Responses were scored on an 8-point scale ranging from 0 days to 7 days. According to the recommendations of the authors of the screening items, the mean of both scales was calculated [52].

#### Consumption of healthful foods

To obtain information on the consumption of healthful foods, adolescents completed a Food Frequency Questionnaire [54]. The adolescents answered the question ‘How often do you normally eat the following foods?’ for 25 food items. Salad, vegetables and fruit represented healthful foods according to the recommendations ‘10 rules of healthful eating’ specified by the German Society of Nutrition [55]. The consumption of salad, vegetable and fruit was rated on a 7-point Likert-type scale (‘never’, ‘approximately one time per month’, ‘several times a month’, ‘approximately one time a week’, ‘several times a week’, ‘every day’, ‘several times a day’). The mean was calculated of the ratings of salad, vegetable and fruit, representing the consumption of healthful foods.

### Data analysis

Statistical analyses were performed with IBM SPSS Statistics version 22 (IBM Corp., NY, USA). For all variables, less than 5% of values were missing. Missing data were imputed using the expectation maximization algorithm after checking that missing values were completely at random using Little’s MCAR test [56]. Item distributions were inspected for multivariate normality. Skewness and excess of all items were below the thresholds of 2 and 7, respectively, as suggested by Curran, West, and Finch [57].

Structural equation modelling was performed with IBM AMOS 22 (IBM Corp., NY, USA) using maximum likelihood estimation to test the hypothesized sequence. The commonly recommended fit indices χ^2^/df, CFI, SRMR and RMSEA were used to assess the goodness of fit. A good fit is indicated by 0 ≤ χ^2^/df ≤ 2, .97 ≤ CFI ≤ 1, 0 ≤ SRMR ≤ .05 and RMSEA ≤ .05, and an acceptable fit was indicated by 2 < χ^2^/df≤ 3, .95 ≤ CFI < .97, .05 < SRMR ≤ .10 and .05 < RMSEA ≤ .08 [58].

The bootstrapping procedure was used to obtain estimates of total, direct and indirect effects. For calculating bias-corrected 95% confidence intervals, 5000 bootstrapping iterations were requested [59]. Standardized values were used to interpret the results.

## Results

### Descriptive statistics

According to the research question and the hypotheses, data from those families where mother, father and adolescent completed the questionnaires, lived in the same household and where the adolescent was aged between 12 and 16 years old were included (N = 198). Means and standard deviations of families’ perceptions of FHC, adolescents’ intrinsic motivation, physical activity and consumption of healthful food are presented in Table 1. Family scores on the FHC_agg_-NU Scale ranged from 3.8 to 8.5 (M = 6.7, SD = 0.9). The scores of the FHC_agg_-PA Scale ranged from 1.2 to 8.9 (M = 4.9, SD = 1.4). These values did not differ between educational levels (low vs. high level of education) of mothers (FHC_agg_-NU: t(189) = 0.49, p = .63; FHC_agg_-PA: t(189) = .39, p = .70) and fathers (FHC_agg_-NU: t(190) = 0.19, p = .85; FHC_agg_-PA: t(139.38) = 0.94, p = .35). In contrast, the FHC nutrition differed between adolescents’ school levels (t(196) = 2.73, p = .007) and gender (t(196) = 2.30, p = .02). The FHC_agg_-NU score was significantly higher in families with an adolescent visiting the highest school level and in families were the participating child was a girl. Furthermore, girls ate more fruits (t(122.84) = 4.04, p < .001) and were more intrinsically motivated to choose healthful foods (t(128.92) = 2.98, p = .003) than boys. Adolescents attending the highest school level (M = 4.04, SD = 1.55) were slightly more physically active than adolescents attending other types of schools (t(193) = 1.99, p = .048). There were no differences in these variables regarding parents’ educational levels (high vs. low level).

**Table 1:** Descriptive statistics and Pearson correlation coefficients for study variables.

		Sociodemographic parameters								Pearson correlation coefficients (p-values)				
		Gender child		School level child		Educational level mother		Educational level father						
		girls	boys	high	low	high	low	high	low					
Variables		M (SD)	M (SD)	M (SD)	M (SD)	M (SD)	M (SD)	M (SD)	M (SD)	2	3	4	5	6
1	PA *child* ^*1)*^	3.90 (1.46)	3.95 (1.76)	**4.04 (1.55)**	**3.50 (1.63)**	3.87 (1.50)	3.97 (1.67)	3.81 (1.63)	4.06 (1.54)	.43 (< .001)	.31 (< .001)	.17 (.02)	.23 (.001)	.13 (.06)
2	intrinsic motivation PA *child*	2.40 (0.68)	2.24 (0.73)	2.38 (0.70)	2.21 (0.71)	2.44 (0.58)	2.29 (0.70)	2.32 (0.73)	2.35 (0.68)		.48 (< .001)	.16 (.02)	.33 (< .001)	.25 (< .001)
3	FHCagg-PA *family*	5.06 (1.48)	4.73 (1.23)	5.00 (1.34)	4.67 (1.53)	4.84 (1.37)	4.97 (1.43)	4.83 (1.26)	5.04 (1.56)			.14 (.05)	.32 (< .001)	.48 (< .001)
4	dietary behavior *child* ^*2)*^	**12.80 (2.14)**	**11.70 (2.99)**	12.54 (2.30)	11.73 (3.27)	12.45 (2.50)	12.31 (2.47)	12.44 (2.64)	12.23 (2.55)				.41 (< .001)	.36 (< .001)
5	intrinsic motivation HE *child*	**2.40 (0.68)**	**2.24 (0.73)**	2.06 (0.64)	1.92 (0.61)	2.03 (0.61)	2.03 (0.63)	2.06 (0.64)	1.98 (0.62)					.49 (< .001)
6	FHCagg-NU *family*	**6.48 (0.95)**	**6.17 (0.89)**	**6.45 (0.91)**	**6.02 (0.97)**	6.39 (0.99)	6.32 (0.91)	6.37 (0.95)	6.34 (0.94)					

*Note*. FHCagg-PA: Family health climate physical activity; FHCagg-NU: Family health climate nutrition; intrinsic motivation PA: intrinsic motivation to engage in physical activity; intrinsic motivation HE: intrinsic motivation of healthful eating; PA: days per week with more than 60 minutes of physical activity; dietary behavior: consumption of healthful food (salad, vegetable, fruit); significant group effects are indicated in bold. 1) data missing for three adolescents, 2) data missing for eight adolescents.

### Relationship between FHC, adolescents’ intrinsic motivation and adolescents’ physical activity and dietary behavior

Bivariate correlations between specific FHCs (FHC-PA and FHC-NU) within the families and adolescents’ physical activity and consumption of healthful food as well as intrinsic regulation of both behaviors are shown in Table 1. The FHC_agg_-PA significantly correlated with adolescents’ intrinsic exercise motivation and with adolescents’ engagement in physical activity. FHC_agg_-NU significantly correlated with adolescents’ intrinsic motivation of healthful food choice and consumption of healthful foods. Intrinsic motivation to exercise was related to physical activity and intrinsic motivation of healthful eating correlated with the consumption of salad, vegetables and fruit. Besides these ‘within-behavior’ correlations, positive and statistically significant ‘cross-behavioral’ correlations were found (Table 1). Bivariate correlations for girls and boys were shown in Table 2. The correlations do not differ significantly depending on adolescents’ gender.

**Table 2:** Correlations between FHC, intrinsic motivation and behaviour for girls and boys.

	r (p, n)					
	1	2	3	4	5	6
PA *child*		.36 (< .001, 119)	.29 (< .001, 119)	.17 (.071, 115)	.20 (.031, 119)	.08 (.41, 119)
intrinsic motivation PA *child*	.54 (< .001, 76)		.46 (< .001, 120)	.06 (.538, 116)	.27 (.003, 120)	.22 (.014, 120)
FHCagg-PA *family*	.35 (.002, 76)	.50 (< .001, 78)		.05 (.604, 116)	.20 (.029, 120)	.46 (< .001, 120)
dietary behavior *child*	.25 (.037, 72)	.09 (.435, 74)	.12 (.323, 74)		.34 (< .001, 116)	.33 (< .001, 116)
intrinsic motivation HE *child*	.28 (.015, 76)	.37 (< .001, 78)	.46 (< .001, 78)	.33 (.004, 74)		.44 (< .001, 120)
FHCagg-NU *family*	.23 (.049, 76)	.25 (.028, 78)	.50 (< .001, 78)	.31 (.008, 74)	.53 (< .001, 78)	

*Note*. FHCagg-PA: Family health climate physical activity; FHCagg-NU: Family health climate nutrition; intrinsic motivation PA: intrinsic motivation to engage in physical activity; intrinsic motivation HE: intrinsic motivation of healthful eating; PA: days per week with more than 60 minutes of physical activity; dietary behavior: consumption of healthful food (salad, vegetable, fruit). Above diagonal: correlation coefficients for girls; below diagonal: correlation coefficients for boys.

### Conceptual model of family health climate, intrinsic motivation and adolescent physical activity and dietary behavior

**Fig 2:**
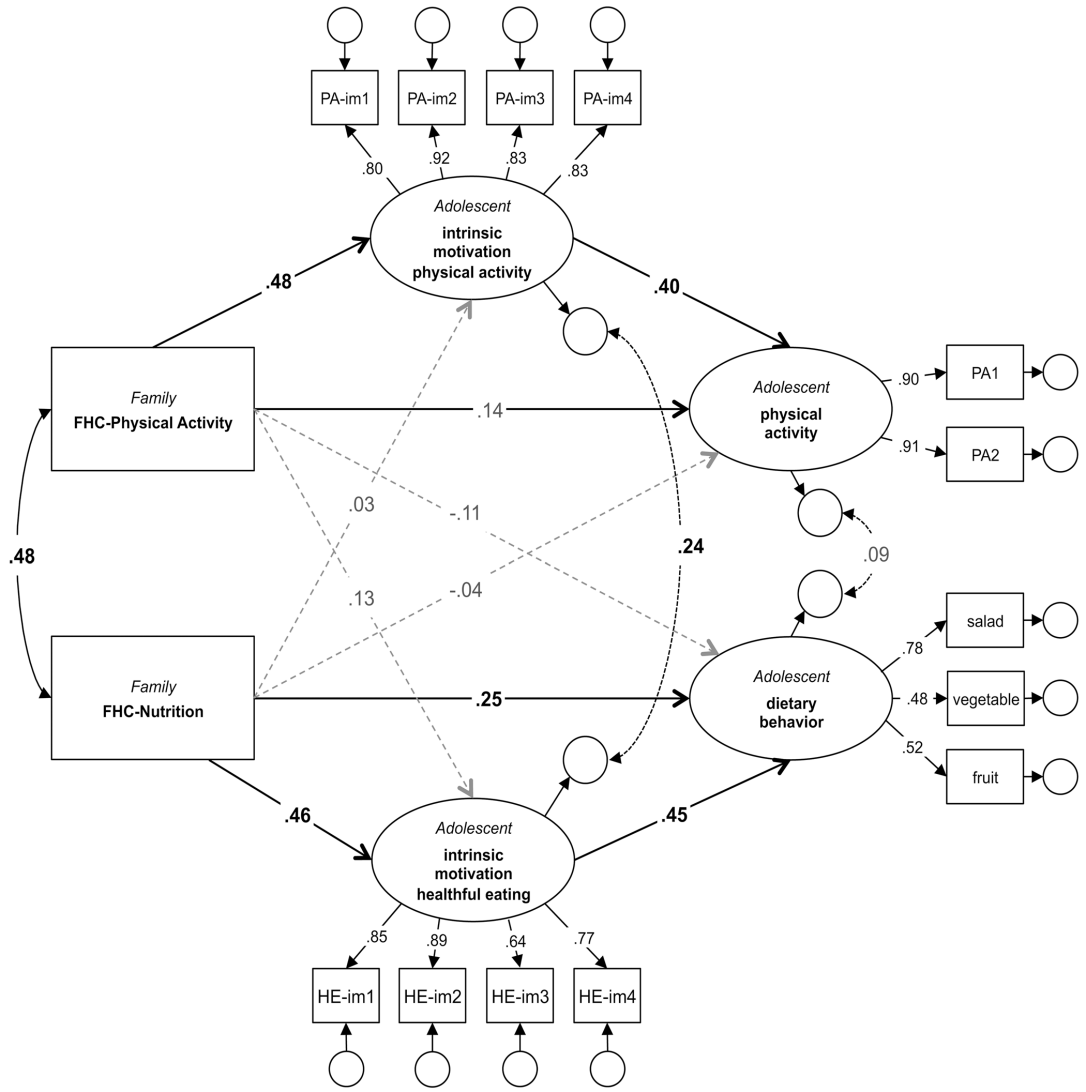
Model of family health climate, intrinsic motivation and adolescent physical activity and dietary behavior. Significant paths are in bold.

A structural equation model reflecting the conceptual model of family health climate, intrinsic motivation and adolescent’s physical activity and dietary behavior (Fig 1) was specified. In addition to the assumed paths the full model includes all cross-behavioral paths (Fig 2). This model showed a good fit to the observed data (χ^2^ = 106.46, df = 79, p = .02; χ^2^/df = 1.35; CFI = .98; SRMR = .05; RMSEA = .04, CI .02/.06, p = .72) and explained 23% of the variance in adolescents’ physical activity and 32% of the variance in the adolescents’ consumption of healthful food.

A cross-behavioral correlation was observed at the environmental level. The more positively the FHC_agg_-PA was perceived by the family, the higher they rated the FHC_agg_-NU (r = .48, p < .001). However, the cross-behavioral paths from specific FHCs to physical activity and dietary behavior, respectively, were not significant. FHC-PA was not significantly related to intrinsic motivation of healthful eating (β = .13, 95% CI = -.001 to .27, p = .05) or consumption of healthful food (β = -.11, 95% CI = -.30 to .05, p = .16). Similarly, FHC-NU was not associated with intrinsic exercise motivation (β = .03, 95% CI = -.13 to .18, p = .75) or weekly amount of physical activity (β = -.04, 95% CI = -.21 to .15, p = .73). Instead, the residuals of intrinsic motivation of healthful eating and intrinsic exercise motivation correlated (r = .24, p = .006), and hence the intrinsic motivation of both health related behaviors shared unexplained variance. While physical activity and dietary behavior were found to co-vary on the environmental and the motivational level, they did not co-vary on the behavioral level (r = .09, p = .39).

### Hypothesis 1 –The FHC is associated with adolescents’ health behavior

The family environmental factors are positively related to adolescent’s health behavior. The more positively the family perceived the FHC concerning physical activity, the higher the adolescents rated their physical activity. The standardized total effect of families’ FHC_agg_-PA on the amount of children’s physical activity per week was .34 (95% CI = .17 to .50, p < .01). A similar pattern was found for dietary behavior. Adolescents’ consumption of fruit, vegetables and salad was significantly predicted by family’s perception of the FHC-NU (standardized total effect .45, 95% CI = .27 to .60, p < .01).

### Hypothesis 2 –The FHCs affect adolescents’ health behavior via adolescents’ intrinsic motivation

While the direct path from family’s FHC_agg_-PA to adolescents’ physical activity was not significant (β = .14, 95% CI = -.05 to .33, p = .16), there was a significant indirect effect of the FHC-PA via adolescents’ intrinsic exercise motivation on their physical activity (β = .19, 95% CI = .11 to .32, p < .01). There was a significant direct effect of families’ FHC_agg_-NU on adolescents’ food consumption (β = .25, 95% CI = .03 to .46, p = .03) and an additional indirect effect via adolescents’ intrinsic motivation to eat healthy (β = .21, 95% CI = .11 to .36, p < .001). As assumed, intrinsic motivation mediated the effect of the FHC on adolescents’ physical activity and dietary behavior.

## Discussion

This study aimed to gain insight into the underlying mechanism of family influences on adolescents’ health behavior considering individual’s motivation as a mediator of the effect of a family environmental factor. This study demonstrated that the family environment, operationalized by the FHC [33], is related to adolescents’ physical activity and healthful food choice in everyday life directly and indirectly via adolescents’ intrinsic motivation.

### FHC, intrinsic motivation and health behavior

The FHC-PA affected physical activity behavior and the FHC-NU affected dietary behavior of adolescents. The absence of cross-behavioral effects indicates that specific factors in the family environment are related to specific health behaviors such as physical activity or healthy diet.

#### Mediating role of intrinsic motivation

The FHC indirectly affected health behavior via adolescent’s motivation for both diet and physical activity. As intrinsic motivation is an individual determinant of long-term health enhancing behavior [43, 44], the relevance of linking family environmental and individual factors becomes evident. The results emphasize the relevance of the mediating role of intrinsic motivation for adolescent health behavior. Many cross-sectional and longitudinal studies have examined family or more precisely parental influences on children’s and adolescents’ health behavior [11]. Other studies have examined cognitive variables such as self-efficacy as mediators of parental behaviors [60, 61]. However, the role of individuals’ motivation as a mediating factor has rarely been considered [29].

Several studies [62–66] using the Self-Determination Theory (SDT) framework examined the role of a need-supportive social environment and evaluated the social environment mostly regarding need satisfaction from the children’s or adolescents’ point of view (mostly perceived autonomy support). This evaluation has been linked to autonomous motivation, cognitions (e.g. intentions, attitudes) and health behaviors (exercise, diet). The present study did not focus on the link between FHC and satisfaction of adolescent’s needs. Therefore, further studies should examine mechanisms underlying the association of FHC and intrinsic motivation, for instance, by considering the satisfaction of the needs for autonomy, relatedness, and competence. Although the mentioned studies did not focus on the role of autonomous motivation as a mediating factor between a family environmental factor and youth’s health behavior, their results indicated the relevance of considering this pathway.

Rutten, Boen and Seghers [67] investigated the role of children’s autonomous motivation as a mediator between environmental factors and pedometer-determined physical activity and found that children’s autonomous motivation (measured by the intrinsic and identified motivation subscales of the BREQ-2) [50] mediated the effect of children’s perceived exercise-related autonomy support and parental activity-related logistic support. Besides the study of Rutten et al. [67], few studies have linked parental behaviors (support, modelling etc.) or environmental factors (rules, availability of foods, family meals etc.) to SDT-based mediating factors. To the best of our knowledge there are no studies investigating the underlying mechanisms of family environmental factors in the nutritional context or integrating both health behaviors.

#### FHC and different health behaviors

The results indicate that consumption of healthful foods and engagement in physical activities are differently affected by the family environment. While for physical activity only the indirect path via individual’s motivation was significant, adolescents’ dietary behavior was directly and indirectly affected by FHC-NU. The FHC-NU may be related to external factors that may have a direct impact on everyday food consumption such as the availability of specific foods [33], frequency of family meals [24] or specific rules [68].

Different motivational profiles may be useful for different behaviors such as physical activity and dietary behavior. Similarly to studying in the educational context, eating healthfully is not inherently enjoyable, especially for children and adolescents. Instead, healthful eating must be internalized within people’s broader goals and values. Therefore, the role of introjected, identified and integrated motivation should receive greater attention in this context [69].

#### Specific and general family environmental factors

Previous studies investigated the clustering of physical activity and healthful eating on an individual behavioral level [37] and regarding parenting practices [70]. This study extended these previous findings to the environmental level shown by the assumed covariance between both specific climates. The covariance between the two specific FHCs suggests the existence of a higher level FHC. This general FHC–representing a general family environmental factor–is responsible for the clustering of physical activity and healthful dietary behavior.

The existence of a higher level FHC is in agreement with the finding of an apparent higher level intrinsic motivation. Intrinsic exercise motivation and intrinsic motivation for healthful eating shared unexplained variance. According to the hierarchical model of intrinsic and extrinsic motivation (HMIEM) [71], a higher level intrinsic motivation should be affected by higher level social factors suggesting that higher level intrinsic motivation may be affected by a general FHC. Moreover, the HMIEM states that the motivation at the contextual level (engagement in physical activity and healthful food choice) is affected by higher level motivation. For instance, Pelletier and Dion [72] have shown that general self-determination is positively associated with more autonomous regulation of eating behaviors and negatively associated with more controlled eating regulation. Furthermore, Mata et al. [38] showed that general self-determination as well as intrinsic exercise motivation predicted eating regulation. Beyond the interplay between different levels of motivation, motivation is also interrelated across different behaviors, which is in agreement with the residual covariance observed in our study.

### Implications for health promoting interventions

Understanding the underlying mechanisms and the interplay between individual and environmental factors affecting children’s and adolescents’ health behavior is crucial for developing health promoting interventions. The important role of parents has been previously shown [17, 73, 74], yet previous studies have focused on providing information to parents on how to influence their children’s behavior, for instance by adjusting their parenting behaviors. The results of our study indicate that higher level family determinants should be included in conceptual models which is in agreement with the family-as-a-system approach [31]. Addressing family as a whole implies to equally involve family members as well as their interactions and aims to create a healthy family environment. A healthy family environment should be reflected in individuals’ perception of the Family Health Climate. This more holistic view of the family is expected to allow tailoring interventions to the family environment to address relevant contextual factors (for example communication related to health and health behaviors, awareness of behaviors, support between all family members) [75]. Setting collective goals or sharing and comparing specific health behaviors such as snacking are strategies, which have been used by recent studies to enhance the presence of health issues in everyday family life [76, 77].

### Strength and limitations

A major strength of our study is the use of a family level variable and the availability of data of both parents (perception of the FHC of both mother and father) and their child (perception of the FHC, intrinsic motivation, health behavior). Using only the child’s rating of the environment could be problematic because this perception could be biased and related to the behavior and related cognitions. In contrast, the aggregated FHC scores used in this study reflect the perception of all three persons, which is a more representative measure of the environment. The use of a multiple-informant approach enhances the validity and reduces the problem of rater bias [78]. Further studies should focus on interpersonal agreement and its moderators.

This study was based on cross-sectional data. The tested model was based on theoretical considerations, and the assumed causalities should be verified by longitudinal studies and experiments. We assessed physical activity and dietary behavior using short self-assessment questionnaires. It remains unknown if data based on objective measurements of physical activity such as accelerometers would have resulted in different associations. The study may have suffered from a relatively low response rate (20%), which may have biased the results. Moreover, the sample was better educated than the average German population [79] possibly limiting the generalizability of the findings. Therefore, replicating the results in other samples or cultures is desirable. We did not observe differences due to educational levels of adolescent, mother and father in the variables that are integrated in the models. The structural equation models were not controlled for potential confounding variables, and hence further studies should consider potential moderators of the relationship between FHC, motivation and health behavior such as family structure, children’s age or gender. Finally, we informed students and parents that the questionnaires should be completed individually. Because we were not able to control this aspect, we carefully checked the plausibility of the data within families in the data clearing process.

## Conclusions

The results of our study showed that intrinsic motivation mediates the influence of the family environment on adolescents’ physical activity and diet. These results emphasize the relevance of the Family Health Climate. This construct appears to be an important aspect of the family environment in the development and maintenance of health behaviors among adolescents. Moreover, considering physical activity and dietary behavior simultaneously revealed potentially different underlying mechanisms for healthful eating than for being physically active. Intrinsic motivation may play a stronger mediating role for being physically active than for healthful eating.

## Supporting Information

S1 Checklist STROBE Checklist for cross-sectional studies. (PDF)

S1 Dataset Dataset underlying the findings in the study. (SAV)

## References

[1] Janssen I. Physical Activity Epidemiology In: Acevedo EO, editor. The Oxford Handbook of Exercise Psychology: Oxford University Press; 2012 pp. 9–34.

[2] Nielsen SJ, Rossen LM, Harris DM, Odgen CL. Fruit and vegetable consumption of U.S. Youth, 2009–2010. NCHS Data Brief 2014; 156(156):1–8. 25027507

[3] Walther J, Aldrian U, Stuger HP, Kiefer I, Ekmekcioglu C. Nutrition, lifestyle factors, and mental health in adolescents and young adults living in Austria. Int J Adolesc Med Health 2014; 26(3):377–386. 10.1515/ijamh-2013-0310 24803606

[4] Jacka FN, Kremer PJ, Berk M, de Silva-Sanigorski AM, Moodie M, Leslie ER et al A prospective study of diet quality and mental health in adolescents. PLoS One 2011; 6(9):e24805 10.1371/journal.pone.0024805 21957462 PMC3177848

[5] Johnson L, Mander AP, Jones LR, Emmett PM, Jebb SA. Energy-dense, low-fiber, high-fat dietary pattern is associated with increased fatness in childhood. Am J Clin Nutr 2008; 87(4):846–854. 18400706 10.1093/ajcn/87.4.846

[6] Bandura A. Human agency in social cognitive theory. Am Psychol 1989; 44(9):1175–1184. 2782727 10.1037/0003-066x.44.9.1175

[7] Sallis JF, Owen N, Fisher EB. Ecological models of health behavior In: Glanz K, Rimer BK, Viswanath K, editors. Health behavior and health education: theory, research, and practice: John Wiley & Sons; 2008 pp. 465–485.

[8] Sallis JF, Nader PR. Family determinants of health behaviors In: Gochman DS, editor. Health behavior: Emerging research perspectives. New York, NY US: Plenum Press; 1988 pp. 107–124.

[9] Sting S. Gesundheit In: Ecarius J, editor. Handbuch Familie: VS Verlag für Sozialwissenschaften; 2007 pp. 480–499.

[10] Campbell TL. Familien und Gesundheit: Zum Stand der Forschung In: Kröger F, Hendrischke A, McDaniel S, editors. Familie, System und Gesundheit. Systemische Konzepte für ein soziales Gesundheitswesen. Heidelberg: Auer; 2000 pp. 225–241.

[11] Lim C, Biddle S. Longitudinal and prospective studies of parental correlates of physical activity in young people: A systematic review. International Journal of Sport and Exercise Psychology 2012; 10(3):211–220.

[12] van der Horst K., Oenema A, Ferreira I, Wendel-Vos W, Giskes K, van Lenthe F et al A systematic review of environmental correlates of obesity-related dietary behaviors in youth. Health Educ Res 2007; 22(2):203–226. 16861362 10.1093/her/cyl069

[13] Edwardson CL, Gorely T. Parental influences on different types and intensities of physical activity in youth: A systematic review. Psychology of Sport and Exercise 2010; 11(6):522–535.

[14] Gustafson SL, Rhodes RE. Parental correlates of physical activity in children and early adolescents. Sports Med 2006; 36(1):79–97. 16445312 10.2165/00007256-200636010-00006

[15] Johnson L, van Jaarsveld C, Wardle J. Individual and family environment correlates differ for consumption of core and non-core foods in children. Brit J Nutr 2011; 105(06):950–959. 21110911 10.1017/S0007114510004484

[16] Lam CB, McHale SM. Developmental patterns and parental correlates of youth leisure-time physical activity. Journal of Family Psychology 2015; 29(1):100–107. 10.1037/fam0000049 25485671

[17] Pugliese J, Tinsley B. Parental socialization of child and adolescent physical activity: a meta-analysis. Journal of Family Psychology 2007; 21(3):331 17874918 10.1037/0893-3200.21.3.331

[18] Kremers SPJ, Brug J, de Vries H, Engels RC. Parenting style and adolescent fruit consumption. Appetite 2003; 41(1):43–50. 12880620 10.1016/s0195-6663(03)00038-2

[19] Pearson N, Atkin AJ, Biddle SJ, Gorely T, Edwardson C. Parenting styles, family structure and adolescent dietary behaviour. Public Health Nutr 2010; 13(08):1245–1253. 19954574 10.1017/S1368980009992217

[20] van der Horst K., Kremers S, Ferreira I, Singh A, Oenema A, Brug J. Perceived parenting style and practices and the consumption of sugar-sweetened beverages by adolescents. Health Educ Res 2007; 22(2):295–304. 16908496 10.1093/her/cyl080

[21] Pinquart M. Associations of general parenting and parent–child relationship with pediatric obesity: A meta-analysis. J Pediatr Psychol 2014; 39(4):381–393. 10.1093/jpepsy/jst144 24756228

[22] Vollmer RL, Mobley AR. Parenting styles, feeding styles, and their influence on child obesogenic behaviors and body weight. A review. Appetite 2013; 71:232–241. 10.1016/j.appet.2013.08.015 24001395

[23] Bauer KW, Neumark-Sztainer D, Fulkerson JA, Hannan PJ, Story M. Familial correlates of adolescent girls' physical activity, television use, dietary intake, weight, and body composition. Int J Behav Nutr Phys Act 2011; 8(1):1–10. 21453516 10.1186/1479-5868-8-25 PMC3078831

[24] Berge JM, Wickel K, Doherty WJ. The individual and combined influence of the “quality” and “quantity” of family meals on adult body mass index. Families, Systems, & Health 2012; 30(4):344. 10.1037/a0030660 PMC3607495 23148980

[25] Boutelle KN, Fulkerson JA, Neumark-Sztainer D, Story M, French SA. Fast food for family meals: relationships with parent and adolescent food intake, home food availability and weight status. Public Health Nutr 2007; 10(01):16–23. 17212838 10.1017/S136898000721794X

[26] Friend S, Fulkerson JA, Neumark-Sztainer D, Garwick A, Flattum CF, Draxten M. Comparing childhood meal frequency to current meal frequency, routines, and expectations among parents. Journal of Family Psychology 2015; 29(1):136–140. 10.1037/fam0000046 25485670 PMC4386688

[27] Hammons AJ, Fiese BH. Is frequency of shared family meals related to the nutritional health of children and adolescents? Pediatrics 2011; 127(6):e1565–e1574. 10.1542/peds.2010-1440 21536618 PMC3387875

[28] Maitland C, Stratton G, Foster S, Braham R, Rosenberg M. A place for play? The influence of the home physical environment on children’s physical activity and sedentary behaviour. Int J Behav Nutr Phys Act 2013; 10:99 10.1186/1479-5868-10-99 23958282 PMC3765081

[29] Brustad RJ. Children’s Motivation for Involvement in Physical Activity In: Acevedo EO, editor. The Oxford Handbook of Exercise Psychology: Oxford University Press; 2012 pp. 385–408.

[30] Welk GJ, Wood K, Morss G. Parental influences on physical activity in children: An exploration of potential mechanisms. Pediatr Exerc Sci 2003; 15:19–33.

[31] Cox MJ, Paley B. Understanding families as systems. Curr Dir Psychol Sci 2003; 12(5):193–196.

[32] Baranowski T. Families and health actions In: Gochman DS, editor. Handbook of health behavior research 1: Personal and social determinants. New York, NY US: Plenum Press; 1997 pp. 179–206.

[33] Niermann C, Krapf F, Renner B, Reiner M, Woll A. Family health climate scale (FHC-scale): development and validation. Int J Behav Nutr Phys Act 2014; 11(1):30 10.1186/1479-5868-11-30 24593840 PMC4015295

[34] Hofstetter H, Dusseldorp E, van Empelen P, Paulussen, Theo W G M. A primer on the use of cluster analysis or factor analysis to assess co-occurrence of risk behaviors. Prev Med 2014; 67C:141–146. 10.1016/j.ypmed.2014.07.007 25036437

[35] Kremers SPJ. Theory and practice in the study of influences on energy balance-related behaviors. Patient Educ Couns 2010; 79(3):291–298. 10.1016/j.pec.2010.03.002 20371159

[36] Leech RM, McNaughton SA, Timperio A. The clustering of diet, physical activity and sedentary behavior in children and adolescents: A review. Int J Behav Nutr Phys Act 2014; 11. 10.1186/1479-5868-11-4 PMC3904164 24450617

[37] Lytle LA, Kelder SH, Perry CL, Klepp K. Covariance of adolescent health behaviors: the Class of 1989 study. Health Educ Res 1995; 10(2):133–146. 10.1093/her/10.2.119-a 10160226

[38] Mata J, Silva MN, Vieira PN, Carraça EV, Andrade AM, Coutinho SR et al Motivational “spill-over” during weight control: Increased self-determination and exercise intrinsic motivation predict eating self-regulation. Health Psychol 2009; 28(6):709 10.1037/a0016764 19916639

[39] Kremers SPJ, de Bruijn G, Schaalma H, Brug J. Clustering of energy balance-related behaviours and their intrapersonal determinants. Psychol Health 2004; 19(5):595–606.

[40] Vallerand RJ. Intrinsic and extrinsic motivation in sport and physical activity In: Tenenbaum G, Eklund RC, editors. Handbook of sport psychology (3rd Ed.). Hoboken, NJ US: John Wiley & Sons Inc; 2007 pp. 59–83.

[41] Vansteenkiste M, Sierens E, Soenens B, Luyckx K, Lens W. Motivational profiles from a self-determination perspective: The quality of motivation matters. J Educ Psychol 2009; 101(3):671–688.

[42] Deci EL, Ryan RM. Motivation, personality, and development within embedded social contexts: An overview of self-determination theory In: Ryan RM, editor. The Oxford handbook of human motivation. New York, NY US: Oxford University Press; 2012 pp. 85–107.

[43] Teixeira PJ, Carraça EV, Markland D, Silva MN, Ryan RM. Exercise, physical activity, and self-determination theory: A systematic review. Int J Behav Nutr Phys Act 2012; 9(1):78. 22726453 10.1186/1479-5868-9-78 PMC3441783

[44] Teixeira PJ, Patrick H, Mata J. Why we eat what we eat: the role of autonomous motivation in eating behaviour regulation. Nutrition Bulletin 2011; 36(1):102–107.

[45] Grolnick WS, Deci EL, Ryan RM. Internalization within the family: The self-determination theory perspective In: Grusec JE, Kuczynski L, editors. Parenting and children's internalization of values: A handbook of contemporary theory. New York, NY US: John Wiley & Sons Inc.; 1997, pp. 135–161.

[46] Deutsche Forschungsgemeinschaft (DFG). Informationen für Geistes—und Sozialwissenschaftler/innen [cited 9 Mar 2015]. Available: http://www.dfg.de/foerderung/faq/geistes_sozialwissenschaften/index.html.

[47] National Science Foundation (NSF). Interpreting the common rule for the protection of human subjects for behavioral and social science research [cited 9 Mar 2015]. Available: http://www.nsf.gov/bfa/dias/policy/hsfaqs.jsp#exempt.

[48] Deutsche Gesellschaft für Psychologie. Ethische Richtlinien der Deutschen Gesellschaft für Psychologie e.V. und des Berufsverbandes Deutscher Psychologinnen und Psychologen e.V. Available: http://www.dgps.de/index.php?id=96422.

[49] Ekvall G. Organizational climate for creativity and innovation. Eur J Work Organ Psy 1996; 5(1):105–123.

[50] Markland D, Tobin V. A modification to the Behavioural Regulation in Exercise Questionnaire to include an assessment of amotivation. J Sport Exercise Psy 2004; 26(2):191–196.

[51] Pelletier LG, Dion SC, Slovinec-D'Angelo M, Reid R. Why do you regulate what you eat? Relationships between forms of regulation, eating behaviors, sustained dietary behavior change, and psychological adjustment. Motiv Emotion 2004; 28(3):245–277.

[52] Prochaska JJ, Sallis JF, Long B. A physical activity screening measure for use with adolescents in primary care. Arch Pediat Adol Med 2001; 155(5):554–559. 10.1001/archpedi.155.5.554 11343497

[53] World Health Organization. Global recommendations on physical activity for health; 2010. 26180873

[54] Winkler G, Döring A. Validation of a short qualitative food frequency list used in several German large scale surveys. Z Ernaehrungswiss 1998; 37(3):234–241. 10.1007/pl00007377 9800314

[55] Deutsche Gesellschaft für Ernährung e.V. Vollwertig essen und trinken nach den 10 Regeln der DGE; 2013 [cited 14 Mar 2014]. Available: http://www.dge.de/modules.php?name=Content&pa=showpage&pid=15.

[56] Little RJA. A test of missing completely at random for multivariate data with missing values. J Am Stat Assoc 1988; 83(404):1198–1202.

[57] Curran PJ, West SG, Finch JF. The robustness of test statistics to nonnormality and specification error in confirmatory factor analysis. Psychological methods 1996; 1(1):16.

[58] Schermelleh-Engel K, Moosbrugger H, Müller H. Evaluating the fit of structural equation models: Tests of significance and descriptive goodness-of-fit measures. Methods of psychological research online 2003; 8(2):23–74.

[59] Hayes AF. Beyond Baron and Kenny: Statistical mediation analysis in the new millennium. Communication Monographs 2009; 76(4):408–420.

[60] Trost SG, Sallis JF, Pate RR, Freedson PS, Taylor WC, Dowda M. Evaluating a model of parental influence on youth physical activity. A J Prevc Med 2003; 25(4):277–282. 10.1016/s0749-3797(03)00217-4 14580627

[61] Peterson MS, Lawman HG, Wilson DK, Fairchild A, van Horn ML. The association of self-efficacy and parent social support on physical activity in male and female adolescents. Health Psychol 2013; 32(6):666–674. 10.1037/a0029129 22888813 PMC3502660

[62] George M, Eys MA, Oddson B, Roy-Charland A, Schinke RJ, Bruner MW. The role of self-determination in the relationship between social support and physical activity intentions. J Appl Soc Psychol 2013; 43(6):1333–1341.

[63] Hagger M, Chatzisarantis NLD., Hein V, Soós I, Karsai I, Lintunen T et al Teacher, peer and parent autonomy support in physical education and leisure-time physical activity: A trans-contextual model of motivation in four nations. Psychol Health 2009; 24(6):689–711. 10.1080/08870440801956192 20205021

[64] Hagger MS, Chatzisarantis NLD., Harris J. From psychological need satisfaction to intentional behavior: Testing a motivational sequence in two behavioral contexts. Pers Soc Psycho B 2006; 32(2):131–148. 10.1177/0146167205279905 16382077

[65] Joussemet M, Landry R, Koestner R. A self-determination theory perspective on parenting. Can Psychol 2008; 49(3):194–200.

[66] Morrison SA, Dashiff CJ, Vance DE. Role of parental autonomy support on self-determination in influencing diet and exercise motivation in older adolescents. Nursing: Research & Reviews 2013; 3.

[67] Rutten C, Boen F, Seghers J. The Relation Between Environmental Factors and Pedometer-Determined Physical Activity in Children: The Mediating Role of Autonomous Motivation. Pediatr Exerc Sci 2013; 25(2):273–287. 23505004 10.1123/pes.25.2.273

[68] de Bourdeaudhuij I. Family food rules and healthy eating in adolescents. J Health Psychol 1997; 2(1):45–56. 10.1177/135910539700200105 22012796

[69] Rutten G, Meis J, Hendriks M, Hamers F, Veenhof C, Kremers S. The contribution of lifestyle coaching of overweight patients in primary care to more autonomous motivation for physical activity and healthy dietary behaviour: results of a longitudinal study. Int J Behav Nutr Phys Act 2014; 11(1):86. 25027848 10.1186/s12966-014-0086-z PMC4132211

[70] Rodenburg G, Oenema A, Kremers SPJ, van de Mheen D. Clustering of diet-and activity-related parenting practices: cross-sectional findings of the INPACT study. Int J Behav Nutr Phys Act 2013; 10(1):36. 23531232 10.1186/1479-5868-10-36 PMC3618009

[71] Vallerand RJ, Ratelle CF. Intrinsic and extrinsic motivation: A hierarchical model In: Deci EL, Ryan RM, editors. Handbook of self-determination research. Rochester, NY US: University of Rochester Press; 2002 pp. 37–63.

[72] Pelletier LG, Dion SC. An examination of general and specific motivational mechanisms for the relations between body dissatisfaction and eating behaviors. J Soc Clin Psychol 2007; 26(3):303–333.

[73] Golan M, Crow S. Parents are key players in the prevention and treatment of weight‐related problems. Nutr Rev 2004; 62(1):39–50. 14995056 10.1111/j.1753-4887.2004.tb00005.x

[74] Kitzmann KM, Dalton WT III, Stanley CM, Beech BM, Reeves TP, Buscemi J et al Lifestyle interventions for youth who are overweight: A meta-analytic review. Health Psychol 2010; 29(1):91–101. 10.1037/a0017437 20063940

[75] Kitzman-Ulrich H, Wilson DK, StGeorge SM, Lawman H, Segal M, Fairchild A. The integration of a family systems approach for understanding youth obesity, physical activity, and dietary programs. Clin Child Fam Psych 2010; 13(3):231–253. 10.1007/s10567-010-0073-0 PMC3293190 20689989

[76] Colineau N, Paris C. Motivating reflection about health within the family: The use of goal setting and tailored feedback. User Modeling and User-Adapted Interaction 2011; 21:341–376.

[77] Schaefbauer C, Kahn D, Le A, Szechowski G, Siek K. Snack Buddy: Supporting Healthy Snacking in Low Socioeconomic Status Families. Proceedings of the 18th ACM Conference on Computer Supported Cooperative Work & Social Computing 2015. 10.1145/2675133.2675180

[78] Bögels S.M., van Melick M. The relationship between child-report, parent self-report and partner report of perceived parental rearing behaviors and anxiety in children and parents. Pers Indiv Differ 2004; 37: 1583–1596.

[79] Statistisches Bundesamt. Zahlen und Fakten: Bildungsstand [cited 3 Aug 2015]. Available: https://www.destatis.de/DE/ZahlenFakten/GesellschaftStaat/BildungForschungKultur/Bildungsstand/Tabellen/Bildungsabschluss.html.

